# Commentary: Ineffectiveness of Commercial Weight-Loss Programs for Achieving Modest but Meaningful Weight Loss: Systematic Review and Meta-Analysis

**DOI:** 10.3389/fpubh.2018.00067

**Published:** 2018-03-06

**Authors:** Amanda Jayne Avery

**Affiliations:** ^1^Division of Nutritional Sciences, University of Nottingham, Nottingham, United Kingdom; ^2^Nutrition, Health and Research, Slimming World, Derby, United Kingdom

**Keywords:** 5% weight loss, effectiveness, obesity, health benefits, commercial weight loss programmes

This commentary refers to a review and meta-analysis investigating weight loss achievements in healthy adults, either overweight or obese, who accessed a commercial weight loss programme (CWLP) (1). This commentary considers how the data has been presented and interpreted, especially given the fact that the 2013 Australian guidelines are very clear that even small amounts of weight loss bring health benefits to individuals with overweight or obesity (2). In addition, the UK National Institute for Health and Clinical Excellence (NICE) 2014 guidelines have perhaps been misinterpreted. The NICE guidelines stated that, for people who are overweight or obese, “the more weight people lose, the greater the health benefits, particularly if someone loses more than 5% of their body weight and maintains this for life” and “weight management programmes can be commissioned if they are likely to lead to an average weight loss of at least 3%, with at least 30% of participants losing >5% of their initial weight” (3). Yet, this review (1) infers CWLPs to be ineffective if participants lost <5% of their initial weight.

In terms of the methodology and approaches for intention-to treat (ITT) analysis employed for each study, the following included, last observation carried forward (LOCF), baseline observation carried forward (BOCF), or non-completer data where it is assumed that the latter will all lose <5%. A pragmatic approach and use of LOCF and BOCF is appropriate, but one question why the latter has been employed as this method does not reflect ITT and some non-completers may achieve a 5% weight loss depending on the length of the programme and also the variability in the definition of a completer between individual studies. For example, in the audit of 1.3 million people who self-referred to Slimming World (SW) while 75.7% of the “completers” lost >5%, so did 16.6% of the “non-completers” (4).

Using ITT analysis, 43% of all the individuals (*n* = 1,443,208) and 63% of programme completers, included in this review, lose 5% or more of their initial body weight thus exceeding NICE guidelines for commissioned weight management programmes. Hence, it is difficult to comprehend how the authors substantiated their argument; “*we conclude that CWLPs frequently fail to produce modest but clinically meaningful weight loss*…”

In addition, the review infers that weight loss outcomes reported are as per programme end and this is not necessarily the case. For example, for the 1.3 million fee-paying participants (4), the audit data is reported 3 months after enrolment. Yet, many of the 436,666 people who were still attending the weekly groups after 14 weeks would have continued to attend and achieved even greater weight losses. Figure 2 therefore has an error in that the data presented on duration of the programme for Stubbs et al. (4) is 3 months and not a self-selected period. For some of the other studies included, participants may have chosen to continue to attend the intervention after the study period had finished. Generally for programmes with a greater duration a greater proportion of participants achieve ≥5% weight loss as would be expected, given the correlation between attendance and weight loss outcomes (5). This correlation is completely overlooked in the discussions within the paper. The review authors did not to include a comparable forest plot showing the data and meta-analysis outcomes for those participants who were classified as having completed the programme relevant to them.

Commenting further on the methodology given, the research objective and specifically the definition of the most comprehensive dataset (larger or smaller, but with longer duration weight loss outcomes), the following observational study could have been identified, selected, and the six-month outcome data presented in the Supplementary Material: Stubbs et al. (6). We are assuming that pregnancy was an exclusion criterion despite not being stated.

While it was pleasing to see that the Cochrane tool was used to determine the level of bias for each of the identified studies, what is not clear is how the “other” bias domains were defined and why “self-selected programme duration”, for example, may have caused a threat to the validity of the primary outcome measures? The risk of bias was considered to be high for the majority of the included studies.

Clearly meal replacements, meal plans (energy restricted), and pre-packaged meal programs offer very different approaches to weight management whether commercially delivered or not, but people with obesity are not a homogenous population and will benefit from options being available to them.

The authors mention that a number of other reviews have considered how commercially delivered programs compare to other types of weight loss intervention, but fail to mention any actual data—are the other options better, equally ineffective, or worse in helping people to achieve modest weight losses? Also how scalable and cost effective are the other options given the magnitude of the obesity epidemic? The discussion does allude to the fact that the commercially delivered programmes do result in greater mean weight losses than other options providing a similar level of intervention in terms of intensity. So perhaps other options are even less effective?

The paper concludes with the statement that the findings, including the high rate of attrition, suggest that many consumers find dietary changes required by commercially delivered programmes to be unsustainable. This statement completely ignores the complexity of obesity and weight management, and the difficulties that some people with obesity encounter and how they will need to engage with any programme on a number of occasions before they are successful in their weight loss journey—whatever success may look like: modest or perhaps clinically meaningful? An integrated management approach may promote better long-term adherence to recommended lifestyle changes (7).

The advantage of the commercially delivered programs is, besides investing heavily in supporting research to offer an evidence-based behavioral change programme to support each individual with obesity, that they are available as when an individual feels ready to make changes to their lifestyle—even if the individuals personal circumstances change somewhere along the journey.

## Author Contributions

The author confirms being the sole contributor of this work and approved it for publication.

## Conflict of Interest Statement

AA in addition to her academic position at the University of Nottingham also receives a salary from Slimming world, a commercial slimming organization, for her role as Consultant Dietitian in weight management.
